# Accuracy of the National Early Warning Score version 2 (NEWS2) in predicting need for time-critical treatment: retrospective observational cohort study

**DOI:** 10.1136/emermed-2024-214562

**Published:** 2025-01-11

**Authors:** Steve Goodacre, Laura Sutton, Gordon Fuller, Ashleigh Trimble, Richard Pilbery

**Affiliations:** 1 Sheffield Centre for Health and Related Research (SCHARR), The University of Sheffield, Sheffield, UK; 2 Emergency Department, Northern General Hospital, Sheffield, UK; 3 Yorkshire Ambulance Service NHS Trust, Wakefield, UK

**Keywords:** clinical assessment, triage

## Abstract

**Background:**

Initial ED assessment can use early warning scores to identify and prioritise patients who need time-critical treatment. We aimed to determine the accuracy of the National Early Warning Score version 2 (NEWS2) for predicting the need for time-critical treatment.

**Methods:**

We undertook a single-centre retrospective observational cohort study. We randomly selected 4000 adults who attended a tertiary hospital ED in England from 1 January 2022 to 31 December 2022 and had NEWS2 routinely recorded on electronic patient records. The first NEWS2 and vital signs were extracted from electronic records. Research nurses selected cases that received a potentially time-critical treatment. Two independent clinical experts then determined whether time-critical treatment was or should have been received using an expert consensus derived list of interventions. We used receiver operating characteristic analysis and calculated sensitivity and specificity at predefined thresholds to evaluate the accuracy of NEWS2 for predicting need for time-critical intervention and, as a secondary outcome, mortality at 7 days.

**Results:**

After excluding 10 patients who received their intervention before NEWS2 recording, 164/3990 (4.1%) needed time-critical treatment and 71/3990 (1.8%) died within 7 days. NEWS2 predicted need for time-critical treatment with a c-statistic of 0.807 (95% CI 0.765 to 0.849) and death within 7 days with a c-statistic of 0.865 (95% CI 0.813, 0.917). NEWS2>4 predicted need for time-critical treatment with sensitivity of 51.8% (95% CI 44.2%, 59.3%) and positive predictive value of 25.8% (95% CI 21.3%, 30.7%). 37 of the 45 patients needing emergency surgery, antibiotics for open fractures, insulin infusion or manipulation of limb-threatening injuries had NEWS2≤4. Patients with NEWS2>4 who did not need time-critical treatment frequently scored maximum points on NEWS2 for their respiratory rate, conscious level or receiving supplemental oxygen.

**Conclusion:**

NEWS2 has limited accuracy for predicting need for time-critical treatment. We have identified time-critical interventions that frequently have low NEWS2 scores and NEWS2 parameters than may overestimate need for time-critical intervention.

**Trial registration number:**

Research Registry 10450

**WHAT IS ALREADY KNOWN ON THIS TOPIC**

- The National Early Warning Score version 2 (NEWS2) is used to identify patients needing time-critical treatment at initial ED assessment, but NEWS2 validation has focused on predicting the risk of death.

**WHAT THIS STUDY ADDS**

- This retrospective observational cohort study of 4000 patients showed that NEWS2 predicted the need for time-critical treatment with limited accuracy (c-statistic 0.807). Half of patients needing time-critical treatment and most patients needing emergency surgery, antibiotics for open fractures, insulin infusion or manipulation of limb-threatening injuries had a low NEWS2 score (0–4).

**HOW THIS STUDY MIGHT AFFECT RESEARCH, PRACTICE OR POLICY**

- ED clinicians can use our data to recognise the limitations of NEWS2 as an initial assessment tool and identify cases where NEWS2 may underestimate or overestimate the need for time-critical treatment.

## Background

 Patients attending the ED can face frequent and sometimes prolonged waits before they receive definitive assessment and treatment. This can result in avoidable harm if urgent treatment is delayed. Initial assessment of patients arriving at the ED aims to reduce this risk by ensuring that patients with time-critical conditions are identified and prioritised.^1 2^

Early warning scores use physiological measurements to produce a composite score reflecting illness severity that can assist initial assessment. In the UK, guidance from the Royal College of Emergency Medicine supports the use of early warning scores as part of initial assessment but advises against their use as a sole measure of acuity.^1^ The Royal College of Physicians (RCP) developed the National Early Warning Score version 2 (NEWS2) to standardise the assessment and response to acute illness in adults and recommends that ED staff use NEWS2 to aid the initial assessment of adult patients.^3^ NHS England has endorsed the use of NEWS2 and provided guidance to support adoption in acute and ambulance settings.^4^

Systematic reviews and meta-analysis have shown that early warning scores have good prediction for mortality but adequate to poor prediction of intensive care or hospital admission.^5^,^8^ Mortality reflects illness severity, including frailty and long-term conditions, but early warning scores need to predict illness acuity—the need for time-critical treatment. Mortality identifies deaths that occurred despite treatment, and may therefore have been inevitable, but does not identify cases where treatment prevented death.^9^ Mortality may therefore fail to identify cases most likely to benefit from time-critical treatment. A recent review of ED acuity assessment tools^10^ identified only one small study that directly measured accuracy for time-critical treatment^11^ and a systematic review that included studies measuring accuracy for time-critical diagnoses.^12^

We aimed to determine the accuracy with which NEWS2 predicts the need for time-critical treatment among adults attending the ED and characterise presentations where NEWS2 has poor accuracy.

## Methods

### Design and setting

We undertook a single-centre retrospective observational cohort study at the Northern General Hospital ED in Sheffield, UK. This is the only adult ED serving the 530 000 population of Sheffield and the adult major trauma centre for the 1.8 million population of South Yorkshire. The ED receives all undifferentiated adult emergencies except ambulance arrivals with stroke or ST-elevation myocardial infarction requiring reperfusion, which are taken directly to specialist services. At initial assessment, nurses record vital signs on the ED information system for all patients considered to be at risk of physiological deterioration, which then generates a NEWS2 score for patients with a complete set of vital signs. Oxygen saturation is recorded using scale 1 unless the patient is known to have hypercapnic respiratory failure. Scale 1 allocates NEWS2 points only according to the severity of hypoxia, whereas scale 2 also allocates points to patients with confirmed hypercapnic respiratory failure if they have oxygen saturation exceeding 92% and are receiving supplemental oxygen. The patient tracking system and electronic record flag any patient with a total NEWS2>4 or a single parameter of 3 as requiring an urgent response and any patient with a total NEWS2>6 as requiring an emergency response, in accordance with RCP guidance.^3^ Patients with minor injuries or primary care complaints are registered with the ED but may then be referred to services located alongside the ED without NEWS2 being recorded. The attending clinician completes a coding form when ED assessment is complete that records standardised ED diagnoses and treatments.

### Case selection

We used routine hospital data to identify all adult (aged 16 or over) ED attendances from 1 January 2022 to 31 December 2022 that had a NEWS2 score recorded and randomly sampled 4000 attendances to account for seasonality, having excluded repeat attendances and patients who had opted out of allowing their data to be used for research. Each eligible attendance was given a random number on an Excel spreadsheet, attendances were sorted in order of the random number and then consecutively selected. We extracted the following routine hospital data: age, sex, ethnicity, the first recorded NEWS2 score, heart rate, respiratory rate, temperature, peripheral oxygen saturation, blood pressure and conscious level, ED diagnoses and treatments, hospital admission, and attendances, admissions and deaths over the following week.

A research nurse reviewed the ED records, the initial inpatient records for admitted patients and hospital discharge summaries of the selected attendances to identify patients who received any treatment that could potentially be considered time critical or suffered an adverse outcome (death or safety incident) that could have been prevented by time-critical treatment. Two independent experts then reviewed hospital records of the patients selected by the nurses and used their clinical expertise to determine whether the treatment fulfilled the definition of being time critical outlined below, with a third expert resolving any disagreements. The experts (SG, AT, GF) had all completed specialist training in emergency medicine. The NEWS2 score was recorded on an observations chart that was not part of the hospital records reviewed by the experts, so outcome adjudication was undertaken by observers who were not aware of the NEWS2 score but could have estimated or calculated the score from the clinical observations.

We also checked all the selected attendances against the ED database of patient safety incidents recorded using the DATIX system to identify any adverse events that could have been prevented by time-critical treatment. The incident reports were used to select any potentially relevant incidents, which were then independently adjudicated by two of the clinical experts.

### Outcomes

The primary outcome, need for time-critical treatment, was defined through the expert consensus process described in the online supplemental appendix. Nine experts in emergency medicine used existing literature and their clinical experience to define 34 interventions as likely to be time critical and provided advice on how the list of interventions should be used in outcome adjudication. The clinical experts then used this list of interventions to determine whether each patient had appropriately received or should have received a time-critical treatment. We included time-critical interventions provided after leaving the ED if the intervention required the patient to be urgently moved to a specialist facility (eg, operating theatre or intensive care) or if the intervention should have been urgently provided in the ED but was delayed or missed.

We excluded cases from the analysis if they received a time-critical treatment before NEWS2 was recorded or received continuing time-critical prehospital treatment in the ED (eg, airway or breathing support that was initiated prehospital and continued in the ED). We included cases that had received time-critical prehospital intervention if the intervention had been completed and NEWS2 was recorded after completion of the prehospital intervention (eg, treated hypoglycaemia).

### Analysis

A medical statistician from the University of Sheffield (LS) undertook all analysis. NEWS2 scores generated by the hospital information system were verified against their constituent elements. The information system only generates a NEWS2 score if all constituent elements are entered and sets limits for the values entered, so there were no missing data in the study population. However, the system allocates a NEWS2 score of 3 for any variable with a zero value. We therefore checked any zero values against the hospital records to ensure that the score of 3 was appropriately allocated (eg, unrecordable temperature due to hypothermia or unrecordable blood pressure due to shock) and imputed the next available measurement if the zero value appeared to be due to equipment failure.

We calculated a kappa statistic with a 95% CI to determine the agreement between the two experts adjudicating the primary outcome. We undertook receiver operating characteristic (ROC) analysis to determine the discriminant value of NEWS2 for predicting the need for time-critical treatment across varying thresholds.^13^ We repeated this analysis using a secondary outcome of death within 7 days and a composite secondary outcome of death within 7 days or need for time-critical treatment, and tested the hypothesis that NEWS2 prediction differs between time-critical treatment and death. We calculated the sensitivity, specificity, positive predictive and negative predictive values (with 95% CIs calculated using the Wilson score method^14^) for thresholds of NEWS2>4 and NEWS2>6. We repeated this analysis with the score classified as being above the threshold if any NEWS2 parameter equals 3, in accordance with RCP guidance.^3^ Finally, we described the characteristics of the ‘false negative’ cases that had NEWS2≤4 and needed time-critical treatment, and the ‘false positive’ cases that had NEWS2>4 but did not need time-critical treatment.

We estimated that a sample size of 4000 would include 200 cases with the primary outcome, based on the subjective judgement of experienced clinicians working in the ED (SG and GF). This sample would give an estimated 95% CI of 0.73 to 0.81 for an assumed c-statistic of 0.77,^13^ based on a previous similar study.^15^

### Patient and public involvement

Sheffield Emergency Care Forum is a patient and public representative group with an interest in emergency care that has extensive experience of involvement in emergency care research.^16^ Two members of the forum joined the research team and were specifically responsible for reviewing the list of time-critical interventions to ensure that it reflected public values and would not discriminate against any patient group.

## Results

Online supplemental figure 1 shows the flow of cases through the study. There were 85 499 first and 41 220 repeat ED attendances in 2022, with 56 145/85 499 (65.7%) first attendances having NEWS2 recorded. The 27 905 patients without NEWS2 at first attendance were relatively young (mean age 44.5 years), with a low admission rate (1911/27 905, 6.8%) and relatively high proportions of minor injuries (14 346/27 905, 46%) and referral to primary care (5423/27 905, 17.4%). We excluded 2689 eligible cases that had opted out of allowing data use and 46 aged <16 years, and then randomly selected 4000 from 53 410 eligible attendances for inclusion.

The research nurses selected 704/4000 cases with possible time-critical interventions for expert review, with 173/704 adjudicated as needing time-critical treatment (κ=0.89, 95% CI 0.86, 0.93). One additional case was identified through safety incident review, and 10 cases were excluded because the time-critical intervention was received before NEWS2 was recorded. Therefore, 164/3990 (4.1%) cases were positive for the primary outcome. There were 71 participants (1.8%) experiencing the secondary outcome of death within 7 days and 195 (4.9%) with the composite secondary outcome. Table 1 shows the characteristics of the included patients and table 2 shows the time-critical interventions received. The most frequent intervention was intravenous antibiotics for infection causing new organ dysfunction or shock (66/164).

**Table 1:** Participant characteristics (whole cohort)

Variable	All (n=3990)
Age (years)	
Mean (SD)	52.0 (22.8)
Median (min, max)	52.0 (16.0, 107)
Sex	
Female	2173 (54.5%)
Male	1817 (45.5%)
Ethnicity	
Asian	285 (7.6%)
Black	164 (4.4%)
Mixed	54 (1.4%)
Other	236 (6.3%)
White	3007 (80.3%)
Not stated	244
Heart rate (beats per minute)	
Mean (SD)	86.6 (19.0)
Median (min, max)	85.0 (18.0, 199)
Respiration rate (breaths per minute)	
Mean (SD)	18.2 (3.25)
Median (min, max)	18.0 (6.00, 66.0)
Oxygen saturation (%)	
Mean (SD)	97.7 (2.42)
Median (min, max)	98.0 (54.0, 100)
Level of consciousness	
Alert	3857 (96.7%)
Confused	86 (2.2%)
Voice	20 (0.5%)
Pain	17 (0.4%)
Unresponsive	10 (0.3%)
Systolic BP (mm Hg)	
Mean (SD)	139 (25.0)
Median (min, max)	137 (52.0, 271)
Diastolic BP (mm Hg)	
Mean (SD)	83.8 (16.7)
Median (min, max)	83.0 (0, 198)
Temperature (°C)	
Mean (SD)	36.6 (0.762)
Median (min, max)	36.6 (29.1, 40.6)
Supplemental oxygen	
Air	3764 (94.3%)
On O_2_	226 (5.7%)

**Table 2:** Treatment received or should have been received in reference standard positive cases

Intervention	Frequency
Intravenous antibiotics for infection causing new organ dysfunction or shock	66
Emergency surgery to avoid death or significant disability, including surgical source control for infection	21
Large-volume intravenous fluid replacement (>2 L within 2 hours or >1 L with central venous monitoring)	19
Any intervention to support ventilation (except supplemental oxygen), other than during sedation for a procedure	18
Administration of blood products for acute blood loss, to allow emergency procedures, in haematological emergencies, or severe anaemia in context of proven acute coronary syndrome	15
Unplanned airway intervention to provide a patent airway, other than during sedation for a procedure	11
Intravenous antibiotics for genuine open fractures	11
Hyperkalaemia or hypokalaemia involving intravenous treatment and cardiac monitoring	10
Insulin infusion as part of treatment protocol for diabetic ketoacidosis or hyperosmolar hyperglycaemic state	8
Antidote for life or disability threatening poisoning	7
Any intervention to support circulation (including CPR), other than intravenous fluids	7
Intravenous fluids and steroids for Addisonian crisis	6
Reduction of limb-threatening fracture or dislocation (including threat to skin, nerve or perfusion)	5
Cardioversion or rate control for life-threatening arrhythmia	3
Primary percutaneous coronary intervention or thrombolysis for myocardial infarction	3
Parenteral treatment for hypoglycaemia	3
Hypertonic saline for hyponatraemia causing a specific neurological disturbance, such as seizures or reduced conscious level	2
Any intervention to achieve haemorrhage control, other than manual pressure or a dressing	2
Intravenous nitrates for acute heart failure	2
Intravenous antibiotics for meningitis or necrotising fasciitis	2
Emergency reperfusion of an ischaemic limb or organ (eg, testicular torsion)	2
Active rewarming for hypothermia	1
Intravenous treatment to lower life or disability threatening hypertension	1
Thrombolysis for massive pulmonary embolism	1
Any other, not previously specified—acyclovir for herpes simplex meningitis	1

CPR, cardiopulmonary resuscitation.

**Figure 1:**
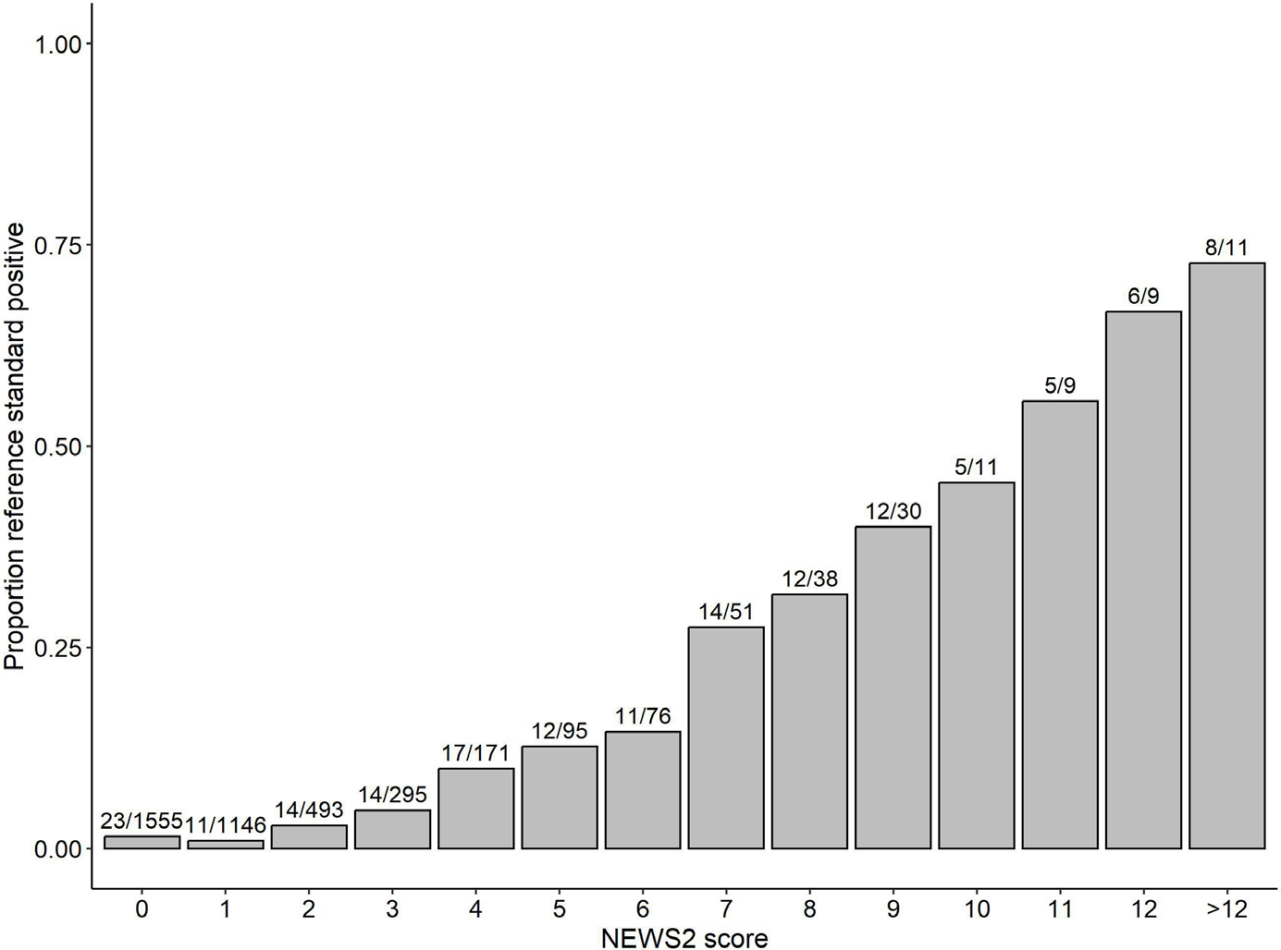
Proportion of cases needing time-critical treatment at each National Early Warning Score version 2 (NEWS2) score.

**Figure 2:**
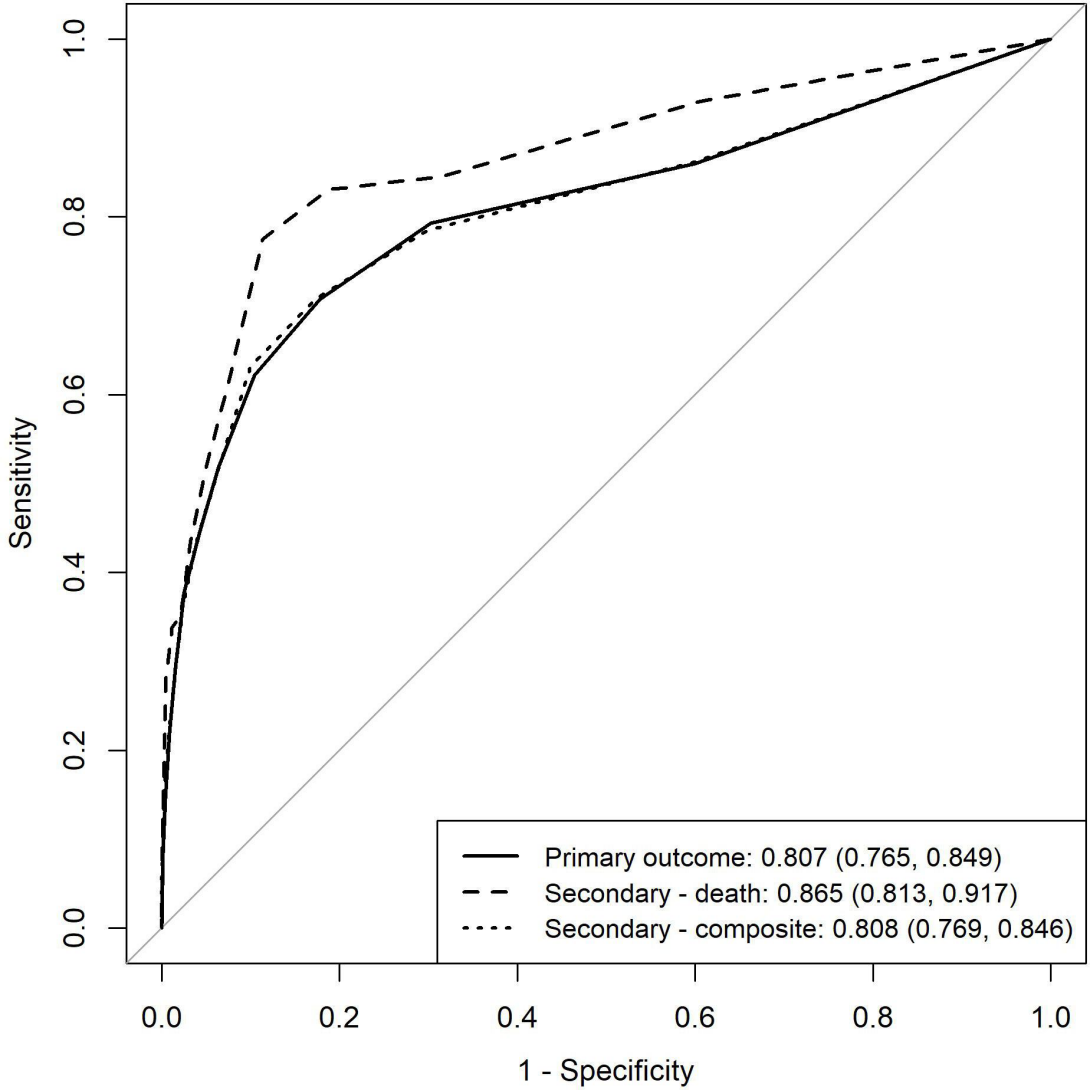
Area under the receiver operating characteristic (AUROC) (95% CI) for National Early Warning Score version 2 (NEWS2) and primary and secondary outcomes.

Figure 1 shows the proportion of cases needing time-critical treatment at each NEWS2 score. The proportion increases with NEWS2 score and exceeds a quarter of cases at NEWS2=7 and half of cases at NEWS2=11, although based on small numbers at higher NEWS2 score. Figure 2 shows the ROC curves for the primary and secondary outcomes. The c-statistic for NEWS2 prediction, the need for time-critical intervention, was 0.807 (95% CI 0.765 to 0.849), which was lower than the c-statistic for death within 7 days (0.865, 95% CI 0.813, 0.917), although the difference was not statistically significant (p=0.09).

Table 3 shows the accuracy of NEWS2 for the primary outcome using recommended thresholds for patient prioritisation. Around half of patients needing time-critical intervention have NEWS2>4 and around a quarter with NEWS2>4 need time-critical intervention. Sensitivity can be improved to around 60%, at the expense of specificity, by including those with any NEWS2 parameter equalling 3 as equivalent to NEWS2>4 (index test positive).

**Table 3:** Diagnostic accuracy statistics (95% CI) for the primary outcome

EWS threshold	TP	FP	FN	TN	Sensitivity	Specificity	PPV	NPV	LR+	LR−
NEWS2>4	85	245	79	3581	51.8%(44.2%, 59.3%)	93.6%(92.8%, 94.3%)	25.8%(21.3%, 30.7%)	97.8%(97.3%, 98.3%)	8.09(6.69, 9.80)	0.51(0.44, 0.60)
NEWS2>4 or parameter 3	98	428	66	3398	59.8%(52.1%, 67.0%)	88.8%(87.8%, 89.8%)	18.6%(15.5%, 22.2%)	98.1%(97.6%, 98.5%)	5.34(4.58, 6.23)	0.45(0.38, 0.55)
NEWS2>6	62	97	102	3729	37.8%(30.7%, 45.4%)	97.5%(96.9%, 97.9%)	39.0%(31.8%, 46.7%)	97.3%(96.8%, 97.8%)	14.91(11.3, 19.69)	0.64(0.57, 0.72)
NEWS2>6 or parameter 3	88	380	76	3446	53.7%(46.0%, 61.1%)	90.1%(89.1%, 91.0%)	18.8%(15.5%, 22.6%)	97.8%(97.3%, 98.3%)	5.40(4.55, 6.41)	0.51(0.44, 0.61)

EWS, early warning system; FN, false negative; FP, false positive; LR, likelihood ratio; NEWS2, National Early Warning Score version 2; NPV, negative predictive value; PPP, positive predictive value; TN, true negative; TP, true positive.

Table 4 shows the time-critical interventions that the false negative cases with NEWS2≤4 needed. Patients needing emergency surgery (18/21), intravenous antibiotics for open fractures (9/11), insulin infusion (5/8) and reduction of limb-threatening injury (5/5) often had NEWS2≤4. The frequency of intravenous antibiotics for infection causing new organ dysfunction or shock among false negative cases reflects the high frequency of this time-critical intervention (14/66).

**Table 4:** Time-critical treatments that patients with NEWS2≤4 needed (n=79)

Intervention	Frequency
Emergency surgery to avoid death or significant disability, including surgical source control for infection	18
Intravenous antibiotics for infection causing new organ dysfunction or shock	14
Intravenous antibiotics for genuine open fractures	9
Administration of blood products for acute blood loss, to allow emergency procedures, in haematological emergencies, or severe anaemia in context of proven acute coronary syndrome	7
Insulin infusion as part of treatment protocol for diabetic ketoacidosis or hyperosmolar hyperglycaemic state	5
Large-volume intravenous fluid replacement (>2 L within 2 hours or >1 L with central venous monitoring)	5
Reduction of limb-threatening fracture or dislocation (including threat to skin, nerve or perfusion)	5
Antidote for life or disability threatening poisoning	5
Hyperkalaemia or hypokalaemia involving intravenous treatment and cardiac monitoring	4
Intravenous fluids and steroids for Addisonian crisis	4
Any intervention to support ventilation (except supplemental oxygen), other than during sedation for a procedure	4
Cardioversion or rate control for life-threatening arrhythmia	3
Primary percutaneous coronary intervention or thrombolysis for myocardial infarction	3
Unplanned airway intervention to provide a patent airway, other than during sedation for a procedure	2
Any intervention to achieve haemorrhage control, other than manual pressure or a dressing	2
Any intervention to support circulation (including CPR), other than intravenous fluids	2
Intravenous nitrates for acute heart failure	2
Emergency reperfusion of an ischaemic limb or organ (eg, testicular torsion)	2
Parenteral treatment for hypoglycaemia	2
Hypertonic saline for hyponatraemia causing a specific neurological disturbance, such as seizures or reduced conscious level	1
Intravenous treatment to lower life or disability threatening BP	1
Intravenous antibiotics for meningitis or necrotising fasciitis	1
Thrombolysis for massive pulmonary embolism	1
Any other, not previously specified	1

CPR, cardiopulmonary resuscitation; NEWS2, National Early Warning Score version 2.

Online supplemental table 1 shows the characteristics of the false negative cases and online supplemental table 2 shows the characteristics of the false positive cases with NEWS2>4 who did not need time-critical treatment. Online supplemental table 3 shows the frequency of NEWS2 parameters scoring maximum points in the false positive cases. Respiratory rate, supplemental oxygen and altered consciousness frequently contributed maximum points in false positive cases.

## Discussion

We found that NEWS2 had limited accuracy in predicting need for time-critical treatment among adult ED attenders. The c-statistic for predicting the need for time-critical treatment was 0.807 (95% CI 0.765 to 0.849). Using a threshold of NEWS2>4 would fail to predict around half of cases needing time-critical intervention and three-quarters of patients with NEWS2>4 would not need time-critical intervention. Most patients needing emergency surgery, antibiotics for open fractures, insulin infusion or manipulation of limb-threatening injuries had NEWS2≤4. Patients with NEWS2>4 who did not need time-critical treatment frequently scored maximum points for respiratory rate, supplemental oxygen or conscious level on NEWS2.

Previous studies have shown that early warning scores have better accuracy for predicting death than for predicting hospital or intensive care admission.^5^,^8^ Meta-analysis of nine studies of NEWS in ED patients reported c-statistics of 0.88, 0.86 and 0.77 for 24-hour, 48-hour and in-hospital mortality, 0.68 for hospital admission and 0.69 for intensive care admission.^5^ A systematic review of 22 studies in acute medical units reported c-statistics of 0.7–0.9 for mortality and <0.6 for intensive care admission.^6^ Our study showed similar prediction of mortality to previous studies.

Few studies have examined accuracy for time-critical treatment. Hong *et al*^11^ compared the Emergency Severity Index and Simple Triage and Rapid Treatment triage tool in predicting 21/233 cases requiring emergent intervention. Hinson *et al*^12^ systematically reviewed triage systems and identified a few studies that evaluated prediction for specific time-critical diagnoses, such as sepsis and myocardial infarction, but none that evaluated accuracy for time-critical interventions.

Our study has shown that it is possible to measure the accuracy of early warning scores for predicting need for time-critical treatment. We developed a reproducible method for adjudicating the primary outcome based on expert consensus and previous literature that we implemented with excellent interobserver agreement. Our study also had low rates of missing data due to the ability of the research team to access hospital records and a sample size that allowed accuracy to be estimated with reasonable precision.

Our study had limitations that need to be appreciated. The outcome adjudicators were not aware of the NEWS2 score but knowledge of the observations that comprise the NEWS2 could have influenced their judgements. We only used the first recorded NEWS2 score, whereas repeated scores may provide more information. The analyses of false negative and false positive cases were based on small numbers of cases. Our definition of time-critical intervention may be contested, with a large proportion of cases involving intravenous antibiotics for infection causing new organ dysfunction or shock, which is based on limited evidence.^17^ Greater use of scale 2 to record oxygen saturation for patients with potential rather than known hypercapnic respiratory failure could reduce the number of false positives arising from low oxygen saturation. Finally, the findings may not be generalisable to EDs with different case mix. Stroke and ST-elevation myocardial infarction requiring reperfusion may be identified as false negative cases in EDs that do not divert these cases to specialist services. NEWS2 may have lower sensitivity for identifying need for time-critical treatment in EDs that receive all such cases. Further research is therefore required to reproduce our findings in other settings.

The implications of our findings are that NEWS2 provides some useful information in identifying need for time-critical treatment, but ED staff should avoid over-reliance on NEWS2 in initial assessment. A substantial proportion of patients needing time-critical treatment will have a low NEWS2 score and most patients with NEWS2>4 will not require time-critical treatment. Concerns about over-reliance on NEWS2 have been raised elsewhere, most notably in a recent coroner’s report to prevent future deaths, which concluded that over-reliance on NEWS2 during ED assessment contributed to the outcome.^18^ We have identified time-critical interventions that NEWS2 predicts poorly and NEWS2 parameters that may overpredict need for time-critical intervention. Further research is required to confirm these findings in other settings and then explore whether NEWS2 can be modified or augmented to improve prediction.

## Supplementary material

10.1136/emermed-2024-214562 online supplemental file 1

10.1136/emermed-2024-214562 online supplemental file 2

## Data Availability

Data are available upon reasonable request.
